# Differences in quality of life in home-dwelling persons and nursing home residents with dementia – a cross-sectional study

**DOI:** 10.1186/s12877-016-0312-4

**Published:** 2016-07-11

**Authors:** Christine Olsen, Ingeborg Pedersen, Astrid Bergland, Marie-José Enders-Slegers, Nina Jøranson, Giovanna Calogiuri, Camilla Ihlebæk

**Affiliations:** Department of Landscape Architecture and Spatial Planning, Section for Public Health Science, Norwegian University of Life Sciences, PO Box 5003, NO-1432 Ås, Norway; Faculty of Health Sciences, Oslo and Akershus University College, Oslo, Norway; Psychology and Educational Sciences, Open University of the Netherlands, Heerlen, Netherlands; Department of Dental Care and Public Health, Hedmark University College, Elverum, Norway; Faculty of Health and Social Work Studies, Østfold University College, Fredrikstad, Norway

**Keywords:** Dementia, Geriatrics, Nursing home, Home-dwelling, ActiGraph, Institutionalization, Sleep, Light exposure, Activity level, Quality of life

## Abstract

**Background:**

Dementia often eventually leads to dependency on others and finally to residential care. However, in Norway about half of the dementia population lives at home, due to individual and political wishes. There is scarce and inconclusive knowledge of how living in a nursing home differs from living at home for persons with dementia (PWDs) with regard to their quality of life (QoL). The first aim of the study was therefore to compare QoL, cognitive and physical functions, social contacts, sleep patterns, physical activity levels, exposure to light, and medication of PWDs in nursing homes and home-dwelling PWDs, and whether living in nursing homes was associated with a lower QoL than living at home for PWDs. A second aim was to examine if possible differences between residencies in QoL were consistent over time.

**Methods:**

The cross-sectional study was based on baseline data from two RCT studies of PWDs. A total of 15 nursing homes with adapted units for PWDs and 23 adapted day care centres for home-dwelling PWDs recruited 78 and 115 participants respectively. Trained nurses scored sociodemographic data, level of dementia (on the Clinical Dementia Rating scale), amount of medication, and QoL (QUALID). Sleep patterns, physical activity levels, and light exposure were measured by actigraphy. A multiple regression analysis was used to test the association between residency and QoL. The association between residency and change in QoL over time was investigated by linear regression analysis of a subsample with follow-up data.

**Results:**

Home-dwelling PWDs showed significantly higher QoL than PWDs in nursing homes. This difference was maintained even after stratifying on the severity of dementia. Home-dwelling PWDs with moderate dementia showed significantly less use of walking aids, more social contact, higher levels of activity and exposure to daylight, and less use of psychotropic medications. The regression model explained 28 % of the variance in QoL in persons with moderate dementia. However, only residency contributed significantly in the model. Residency also significantly predicted negative change over time in QoL.

**Conclusion:**

The study indicated that living at home as long as possible is not only desirable for economic or health political reasons but also is associated with higher QoL for persons with moderate dementia. More studies are needed to investigate how QoL could be increased for PWDs in nursing homes.

## Background

Dementia is among the leading causes of disability and death in the elderly [1, 2], and there are currently 47.5 million people with dementia worldwide [3]. There is no cure for dementia [4], and development of dementia eventually leads to a loss of cognitive and physical functions [5, 6]. This in turn will often lead to total dependency on others and finally to residential care [7]. In Norway, 80 % of nursing home residents suffer from dementia [8].

The incidence of institutionalization varies between European countries [9], and in Norway a higher percentage of the elderly live in nursing homes compared to in other countries [10]. However, it has been estimated that half of the dementia population in Norway still lives at home [11]. Several studies have evaluated risk factors for the institutionalization of the elderly and persons with dementia (PWDs), and older age, cognitive impairment, poor social support, loss of physical function, and use of medication are all factors associated with increased risks of long-term admission to care facilities [9, 12–14].

Residential care can ensure necessary care and safety when PWDs are dependent on help. However, living in a nursing home will affect the lives of PWDs. Many studies have investigated the effect of nursing home environments on different health and behavioural factors [15–18] and the difference between traditional nursing home and small-scale group living [19, 20]. However, few studies have investigated the differences in how living in a nursing home differs from living at home with regard to PWDs’ quality of life (QoL), and they differ in their conclusions [21, 22]. Furthermore, few studies have investigated the QoL of home-dwelling PWDs.

There is no standard definition of QoL among PWDs, and the conceptualizations of QoL vary too [23, 24]. However, the definition by Lawton [25], who states that QoL is a multidimensional concept, which in older adults includes behavioural competence, the objective environment, psychological well-being, and perceived QoL, is frequently used [26].

QoL among elderly persons with dementia is often diminished [27], and poor QoL has been found associated with several of the same risk factors as found for institutionalization, such as low cognitive function, impaired mobility, lack of social activities, major depression, and low performance in activities of daily life [28–31].

Another factor known to affect QoL is sleep disorders [32]. Sleep disruption is common among dementia patients [33], and it has been reported that two-thirds of nursing home residents have sleep disturbance problems [34]. Light exposure, which is an important regulator of circadian rhythm, has been reported to affect activity levels and sleep in nursing home residents [16]. Institutionalized older adults have lower levels of physical activity than elderly persons living in community dwellings [35, 36], and it has been reported that nursing home residents spend up to 94 % of their time sitting or lying down during the daytime [37]. A study of 15 nursing homes revealed that most of the residents spent at least 17 h per day in bed [38]. Another factor that might affect QoL is medication, and some pharmacological treatments have been found associated with lower QoL [39]. In addition to these risk factors, both institutionalization and increased dependency might be negatively related to QoL [21, 40], although for some PWDs admission to long-term facilities might increase their QoL [21].

An important goal in dementia care is to provide for and ensure a good quality of life [40], and it is a common political goal among European governments to enable PWDs to live at home as long as possible [10]. It is therefore important to gain more knowledge of QoL and known associated risk factors in PWDs living at home and living in nursing homes. Furthermore, more studies are needed to investigate the association between residential care and QoL when other risk factors are taken into consideration. The first aim of this article was therefore to compare QoL, cognitive and physical function, social contact, sleep patterns, physical activity, light exposure, and medication in PWDs in nursing homes and home-dwelling PWDs, and examine whether residency was associated with QoL. A second aim was to investigate the association between residency and change in QoL over time.

## Method

Our study was based on data from two RCT studies of PWDs in nursing homes and home-dwelling PWDs in Norway (RCTs registered at ClinicalTrial.gov; NCT02008630 and NCT01998490).

### Recruitment and subjects

In Norway, the municipalities are legally responsible for providing domiciliary and residential care, and the administration of nursing homes and day care centres is organized within the municipalities’ public health services. Most patients receive domiciliary care for as long as possible prior to admittance to a nursing home. The municipalities’ health care and other care services in cooperation with patients’ general practitioners assess patients’ need for residential care. Most nursing homes have both ordinary units and special care units, and often include day care facilities. Special care units are adapted units that usually only house 7–8 PWDs.

For our study, the county development centres for dementia care in three counties in the south-eastern part of Norway were responsible in recruiting nursing homes and day care centres in their municipalities. All nursing homes and day care centres in the three counties were invited to participate in the study, and 15 nursing homes with adapted units for PWDs and 23 adapted day care centres for home-dwelling PWDs were willing to participate.

Each participating institution was asked to recruit between 5 and 8 participants. The inclusion criteria were: aged 65 years or older and either a diagnosis of dementia or a cognitive deficit measured as a score less than 25 on the Mini-Mental State Examination test [41–43].

A total of 209 participants were recruited (88 PWDs living in a nursing home (PWD NH), and 121 home-dwelling PWDs). Due to death or because of withdrawal from the study, 16 participants were excluded from the analyses, which meant that data relating to 193 participants (78 PWD NH and 115 home-dwelling PWDs) were included in the analyses. Home-dwelling participants all took part in a day care centre programme at least once per week. The baseline data collection was carried out in winter–spring 2013 (*N* = 43 (PWD NH = 17, home-dwelling PWDs = 26)), autumn–winter 2013 (*N* = 78 (PWD NH = 31, home-dwelling PWDs = 47)), and spring–summer 2014 (*N* = 72 (PWD NH = 30, home-dwelling PWDs = 42)), mainly due to practical limitations and to avoid seasonal biases. In addition, a subsample consisting of 11 PWDs in nursing homes and 8 home-dwelling PWDs that were randomized to control groups (treatment as usual) was assessed for QoL 6 months after baseline.

### Assessments and procedures for data collection

The participating patients’ primary nurses scored all psychometric assessments and collected information on the participants’ age, gender, educational level, use of walking aids, and social encounters. Before the project, they all participated in mandatory education on the use of the instruments.

**Quality of life (QoL)** was measured using the Norwegian version of the Quality of Life in Late-stage Dementia (QUALID) scale [44, 45]. The proxy rating scale consists of 11 items that are rated on a five-point scale. The items are rated by frequency of occurrence, comprising both positive and negative dimensions of concrete and observable mood and performance. Scores are summed to range from 11 to 55, Cronbach’s alpha = .815 (nursing home = .764, home-dwelling = .719). A lower score indicates a higher quality of life.

The **Clinical Dementia Rating (CDR)** scale, is a 5-point scale used to assess six domains of cognitive and functional performance relating to dementia [46–48]. CDR staging is a valid substitute for a dementia assessment among nursing home residents when rating dementia and determine the severity of dementia [47, 48]. A CDR global score of 0 implies no cognitive impairment, 0.5 = very mild dementia, 1 = mild dementia, 2 = moderate dementia, and 3 = severe dementia. Before the analyses, the CDR scores were recoded into three groups: mild (0, 0.5 and 1), moderate (2), and severe dementia (3).

***Actigraphy*** (ActiSleep+, ActiGraph, Pensacola, US) was used to measure sleep patterns, physical activity levels, and light exposure. ActiSleep + is a validated 3-axis accelerometer, which has approximately the shape and size of a wrist watch and delivers advanced data about the wearer’s movements over time and their exposure to light. The use of actigraphy for monitoring sleep is validated [49], also for dementia patients [50]. The ActiSleep + was worn on the left wrist continuously for 7 days (epoch-length 1 min). Participants were free to remove the ActiSleep + device but were encourage not to do so. Relatives and caregivers were instructed to encourage the participants to put it back on if it had been removed. Before the measurements started, ActiSleep + was introduced orally, visually, and in written form to the participants by their primary nurse, as well as by their relatives and caregivers.

The actigraphy data were processed using the Scoring and Sleep functions of ActiLife, software Version 6.11.2 (ActiGraph, Pensacola, USA), after applying the Wear Time Validation tool. Days with more than 8 h recorded were included in the further analyses in order to ensure that the activity pattern for those days reflected the participant’s typical behaviour pattern. All subjects included in the analysis had at least three valid days and nights.

*Total sleep time* (TST) is the amount of actual sleep during the night-time, measured in hours. The term ‘wake after sleep onset’ (WASO) defines the amount of time spent awake after sleep has been initiated and before final awakening; it sums all wake epochs in minutes. The default algorithm of ActiLife may have problems with analysing the sleep–wake schedule. For that reason, we manually inspected all awakenings and created a new variable called ‘Number of awakenings > 5 min’. By using a minimum awake time of 5 min, we ensured that the number of awakenings were accurate. ‘Sleep efficiency’ was defined as the number of sleep minutes divided by the total number of minutes when the participant was in bed, and was expressed as a percentage. Because of the challenge of identifying a precise bedtime and getup-up time among the home-dwelling population, a default time-in-bed period was arbitrarily set as 23:00 to 06:00 h. Therefore, in our study, sleep efficiency referred to the minutes of sleep within the default time period, and not the patients’ actual time spent in bed, and below this is referred to as the ‘Sleep during night period’.

*Physical activity levels* were calculated using the Freedson Adult Cut Points [51] in ActiLife software, and applying a time filter between 08:00 and 20:00 for each monitored day. ActiLife calculates three activity levels based on the frequency and intensity of the movement. These constitute the measure ‘counts’, which are specified as ‘counts per minute’ (cpm). ‘Sedentary activity level’ is time in percentage with no physical activity (standardized cut point value: 0–99 cpm). ‘Light activity level’ is defined as light intensity activity (standardized cut point value: 100–1951 cpm). Activities in this category could, for example, be standing or household activities. ‘Moderate activity’ (standardized cut point value: 1952–5724 cpm) equates to physical activity, such as walking at 4 km/h. The Freedson Adult Cut Points can also include measures of ‘Vigorous’ activity and ‘Very vigorous’ activity, but these were not used in the study because none of the participants scored any activity at this level. The absolute time (minutes) spent on the different activity levels was subsequently expressed as a percentage of the overall monitoring time.

*Light exposure* was recorded every second and measured in counts, giving ‘lux average counts’, which indicated the participants’ level of exposure to light.

Records of patients’ use of **psychotropic medication** (antipsychotics, antidepressants, anxiolytics, and hypnotics/sedatives) were collected (from no/yes responses) and a score for number of different types of psychotropic medications (0–4) was constructed.

### Ethics

All participants were informed that they could withdraw from the study at any stage. The ActiGraph device worn by the participants did not register what type of activity they engaged in or their localization, and therefore the usage was not considered invasive.

### Analyses

All statistical analyses were performed using IBM SPSS Statistics Version 23.0 for Windows (Armonk, NY: IBM Corp). Cronbach’s alpha was calculated for all sum scores. Group differences between PWD NH and home-dwelling PWDs were tested with one-way ANOVA for continuous variables’ and with chi-square for categorical data. Stratified analyses of the three categories of cognitive level derived from the CDR test were conducted for variables showing significant differences between groups.

A multiple regression analysis was used to test the association between institutionalization and QoL. This model was only tested for PWDs with moderate dementia, due to the low number of persons with mild dementia in nursing homes and low number of persons with severe dementia living at home. Age, gender and variables that were significantly different between the two groups of PWDs in the bivariate analysis, namely social encounters, use of walking aids, physical activity level (moderate), light exposure, and medication, were entered into the analysis in order to control for these factors. Before the analysis, dichotomous variables for walking aids (no/yes) and social contacts (< once per week/≥ once per week) were constructed. Collinearity statistics showed acceptable values (max VIF < 2.3). Heteroscedasticity was not observed.

The association between residency and change in QoL was investigated by linear regression analysis of the subsample with follow-up data (*n* = 19). Change in QUALID was used as the dependent variable, and residency was entered as predictor variable. In order to control for different baseline levels in QoL, the QUALID baseline score was included in the analysis.

## Results

### Group characteristics

There were no significant age, gender, or educational differences between PWDs in nursing homes and home-dwelling PWDs (Table 1). Approximately half of the home-dwelling PWDs lived alone (52.2 %), but they had significantly more social contact with their family members and friends than nursing home PWDs. Walking aids were used by a significantly higher number of PWDs living in nursing homes than home-dwelling PWDs (Table 1). Significant differences were observed in the severity of dementia: 9 % PWDs living in nursing homes had mild dementia, 43.6 % had moderate dementia, and 47.4 % had severe dementia. By contrast, the respective percentages for home-dwelling PWDs were 43.5 %, 47.0 %, and 4.3 % (Table 1).

**Table 1:** Demographic data, quality of life (QUALID), and ActiGraph data relating to persons with dementia in nursing homes (PWD NH) and persons with dementia living at home (home-dwelling PWDs)

	PWD NH	Home-dwelling PWDs	*p*-value
Demographic data			
Women (%)	52 (66.7) (*n* = 78)	74 (64.3) (*n* = 115, 1 missing)	0.877
Age Mean (SD)	84.6 (6.50) (*n* = 78)	82.6 (6.84) (*n* = 103, 12 missing)	0.803
Education (%)	*n* = 78	*n* = 115	0.226
Below upper secondary school	40 (51.3)	43 (37.4)	
Upper secondary school	10 (12.8)	21 (18.3)	
Above upper secondary school	12 (15.4)	28 (24.3)	
Missing	16 (20.5)	23 (20.0)	
Quality of life	(*n* = 77)	(*n* = 109)	
QUALID (SD)	24.06 (7.13)	15.99 (4.33)	<0.001
Clinical Dementia Rating (CDR) scale (%)	*n* = 78	*n* = 115	<0.001
Mild	7 (9.0)	50 (43.5)	
Moderate	34 (43.6)	54 (47.0)	
Severe	37 (47.4)	6 (5.2)	
Missing	0 (0)	5 (4.3)	
Walking aids (%)	*n* = 78	*n* = 115	<0.001
None	24 (30.8)	69 (60)	
Walking sticks/Cane/Crutches	7 (9)	19 (16.5)	
Rollator/High walker	37 (47.4)	21 (18.3)	
Wheelchair	9 (11.5)	0 (0)	
Needs support walking	1 (1.3)	0 (0)	
Missing	0 (0)	6 (5.2)	
Social contact with family/friends (%)	*n* = 78	*n* = 115	<0.001
Every day	5 (6.4)	39 (33.9)	
Several times per week	20 (25.6)	48 (41.7)	
Once per week	31 (39.7)	14 (12.2)	
Once every other week	8 (10.3)	2 (1.7)	
Rare	11 (14.1)	5 (4.3)	
Never	0 (0)	0 (0)	
Missing	3 (3.8)	7 (6.1)	
Sleep patterns	(*n* = 71)	(*n* = 105)	
Sleep during night-time, Mean (SD)	75.89 (15.46)	80.01 (11.88)	0.048
Total sleep time in hours, Mean (SD)	5.31 (1.08)	5.60 (0.08)	0.046
WASO in minutes, Mean (SD)	93.24 (59.47)	73.36 (42.48)	0.011
Wake > 5 min	4.67 (2.80)	4.18 (2.12)	0.190
Activity pattern	(*n* = 71)	(*n* = 107)	
Sedentary % (SD)	51.87 (19.78)	43.51 (14.62)	0.001
Light % (SD)	45.78 (17.70)	50.20 (11.69)	0.045
Moderate % (SD)	2.35 (4.31)	6.29 (5.98)	<0.001
Light exposure (lux av. counts)	(*n* = 71)	(*n* = 107)	
	43.63 (51.40)	165.88 (254.36)	<0.001
Psychotropic medication	(*n* = 68)	(*n* = 88)	
Use of (%)	69.1	35.2	<0.001
Number (SD)	1.12 (0.97)	0.43 (0.64)	<0.001

Notes: *p* < 0.05 (level of significance)

### QoL, sleep patterns, physical activity, light exposure, and medication

In the whole sample, PWDs living in nursing homes showed a significantly lower QoL than home-dwelling PWDs (Table 1). They also scored significantly lower on all sleep parameters, except for ‘Wake > 5 min’. PWDs living in nursing homes experienced almost four times less light exposure compared with home-dwelling PWDs. The actigraphy results showed that PWDs living in nursing homes were less active than and showed significantly more sedentary and less active behaviour than home-dwelling PWDs (Table 1). There were also a significant difference in use of psychotropic medication between PWDs living in nursing homes and home-dwelling PWDs, both in the prevalence and number of medications used (Table 1).

Because of the substantial differences in the severity of dementia between the two populations, a stratified analysis on CDR was conducted to look at differences between the two groups with regard to their cognitive levels (Table 2). PWDs living in nursing homes showed a significantly lower QoL for all three categories of severity of dementia and for the category moderate dementia, they had significantly more use of walking aids, less social contact, lower levels of moderate activity, lower levels of light exposure, and higher use of psychotropic medication (Table 2). The same pattern was found for the categories mild and severe dementia, although few differences were found to be significant.

**Table 2:** Quality of life, medication, use of walking aids, social contact, and ActiGraph data stratified on CDR, mean (SD)

	Mild dementia			Moderate dementia			Severe dementia		
	NH	Home-dwelling	*p*-value*	NH**	Home-dwelling	*p*-value	NH	Home-dwelling	*p*-value
Quality of life	18.86 (6.41)	14.89 (3.74)	0.022	21.94 (6.22)	16.66 (4.37)	<0.001	26.95 (6.94)	18.67 (5.82)	0.009
Walking aids (%)	57.1	47.9	.265	70.6	25.5	<0.001	70.3	33.3	0.004
Social contact weekly (%)	42.9	97.9	<0.001	81.8	90.4	0.004	74.3	80.0	0.058
Sleep patterns									
Sleep during night-time (%)	76.68 (16.54)	79.43 (11.52)	0.579	76.77 (15.51)	79.22 (12.84)	0.453	75.95 (14.68)	86.86 (3.76)	0.111
Total sleep time in minutes	322.04 (69.45)	333.61 (48.37)	0.579	322.41 (65.16)	333.07 (53.96)	0.438	318.99 (61.64)	364.81 (15.77)	0.111
WASO*** (minutes)	92.84 (68.17)	75.71 (37.94)	0.323	89.75 (59.95)	75.02 (48.94)	0.242	93.84 (57.87)	53.47 (16.90)	0.135
Wake > 5 min	4.61 (2.78)	4.31 (1.74)	0.694	4.88 (2.61)	4.16 (2.50)	0.229	4.49 (3.04)	3.20 (1.83)	0.363
Activity pattern									
Sedentary (%)	41.44 (21.17)	43.01 (14.27)	0.800	47.63 (16.67)	43.71 (15.14)	0.281	58.06 (20.76)	48.2 (15.52)	0.317
Light (%)	52.55 (17.95)	50.26 (10.8)	0.634	49.74 (14.21)	50.12 (12.17)	0.900	40.62 (19.58)	49.15 (14.35)	0.357
Moderate (%)	6.01 (7.8)	6.73 (6.8)	0.797	2.63 (4.72)	6.17 (5.36)	0.003	1.32 (2.08)	2.65 (2.05)	0.191
Light exposure (lux av. counts)	93.49 (102.91)	142.55 (211.55)	0.551	39.12 (44.39)	171.84 (275.17)	0.010	37.28 (36.63)	300.15 (328.12)	<0.001
Psychotropic medication	1.17 (0.98)	0.54 (0.61)	0.042	1.17 (1.09)	0.32 (0.60)	<0.001	1.06 (0.88)	0.40 (0.89)	0.126

Notes: *p < 0.05 (level of significance); ** nursing home, *** wake after sleep onset

### Regression analysis

The analysis showed that for PWDs with moderate dementia residency was significantly associated with lower QoL after controlling for age, gender, social encounters, use of walking aids, moderate physical activity level, light exposure, and medication. The model explained 28 % of the variance in QoL (Table 3).

**Table 3:** Association between age, gender, use of walking aids, social contacts, activity level, light exposure, medication, and residency with QUALID in persons with moderate dementia (*N* = 61)

Variables	β	p
Age	-.001	.991
Gender	.028	.806
Use of walking aids ^a^	-.014	.922
Social contact ^b^	.139	.266
Moderate activity ^c^	-.070	.604
Light exposure ^c^	-.145	.243
Psychotropic medication ^c^	.090	.487
Residency ^d^	-.394	.023

Notes:

^a^ Use of walking aids: 0 = No, 1 = Yes

^b^ Social contact: 0 = Less than once a week, 1 = Weekly

^c^ High score indicates to high level of moderate activity, high level of exposure to lux, and high use of psychotropic medication

^d^ Nursing home = 0, Home-dwelling = 1

Standardized beta was used

*p* < 0.05 (level of significance)

Adjusted R^2^ .276

R^2^ change .373

When looking at change in QoL over time, residency explained 25 % of the change in QoL among participants with moderate dementia (F(2,16) = 3.993, *p* = 0.039, R^2^ Adjusted = .25). Nursing home PWDs’ mean change in QoL was 1.73, while home-dwelling PWDs’ mean change was −0.38. The analysis shows that both baseline score on QUALID and institutionalization did significantly predict change in QUALID during 6 months (Beta = −0.63, t(19) = −2.66, p < .05) and (Beta = −0.51, t(19) = −2.16, p < .05) (data not shown).

## Discussion

PWDs living in nursing homes showed significantly lower QoL for all three categories of severity of dementia compared to home-dwelling PWDs. In the group with moderate dementia, PWDs living in nursing homes had significantly more use of walking aids, less social contact, lower level of moderate activity, lower levels of light exposure, and a higher use of psychotropic medication. Living in nursing homes was significantly associated with low QoL, even after controlling for several possible confounders, and associations seem to be consistent over time.

Severity of dementia is known to be highly associated with QoL [27, 30]. We found significantly higher prevalence of severe dementia in nursing home residents than in persons living at home, and similar findings have been reported previously [11, 52]. Decrease in cognitive functioning such as loss of abilities in memory, judgment, and abstract thinking will lead to need for assistance in many activities of daily life [53], and residential care might be necessary in order to provide the care needed. However, in our study, PWDs living in nursing homes showed lower QoL than PWDs living at home, even after stratifying for severity of dementia. This finding is in line with that of a small study that compared QoL among PWDs with mild dementia living in nursing home or at home and found a significant difference in QoL and social contact between the two groups [54].

Several other factors showed significant differences between home-dwelling PWDs and PWDs living in nursing homes after stratifying for severity of dementia, and this finding might be associated with the differences in QoL. For PWDs with moderate dementia, we found that those who were home-dwelling had significantly less use of walking aids and higher level of moderate activity than those living in nursing homes. These differences might indicate that even though the degree of dementia was the same, the PWDs living in nursing homes had poorer physical function. Poor physical function and dependency has been found associated with low QoL [55], but is also a predictor for nursing home admission [9, 13, 14, 56]. In our study, PWDs living in nursing homes had a lower frequency of social contact, and lack of social support is also known to be a predictor for institutionalization [7]. The higher use of psychotropic medication found among PWDs living in nursing homes compared with home-dwelling PWDs is in line with previous studies [57, 58]. Halvorsen et al. suggest that such differences in medication could be explained by more prevalent behavioural and psychiatric symptoms (BPSD) in PWD NH [58]. We did not have any measurements on BPSD for the home-dwelling population and therefore could not compare the two groups on these factors. This constituted a weakness in our study, as BPSD is known to affect patients’ quality of life (QoL) [40, 52] and might also be associated with institutionalization [13, 56].

One European study investigating how QoL varied according to living arrangements concluded that there were no clinically significant differences in QoL between PWDs living in nursing homes and home-dwelling PWDs [21]. However, in our study, living in a nursing home was associated with a lower QoL than living at home for persons with moderate dementia, even after controlling for confounding or mediating factors. Being institutionalized might lead to loss of control and lack of autonomy (e.g. when and what to eat, when to sleep, and when to go for a walk), and Heggestad et al. found that many nursing home residents did not feel at home in their unit and missed their former homes [59]. Furthermore, nursing home residents do not often participate in activities and tend to be unoccupied for much of the day [60]. Several studies of institutionalized PWDs have shown that the residents’ needs for meaningful activities are often unmet [15, 17, 61]. In the Netherlands it has been shown that small-scale settings provide better environments for social relationships than traditional nursing home settings, and one study revealed that residents had significantly higher scores on the QoL subscale ‘positive affect’ [62]. However, other studies have found mixed results on the effects of small-scale settings [63], and that residents perceived a more traditional nursing home environment as satisfactory and that being deprived of privacy was not a problem [64].

The longitudinal QoL data from the sub-group with moderate dementia enabled us to take unobserved heterogeneity into account and thereby detect developments or changes in the characteristics of the population. The fact that nursing home residents had a decrease in mean change of QoL, whereas home-dwelling PWDs were stable over a 6-month period supports the findings that living in a nursing home affect QoL negatively.

### Strengths and limitations

This study had several limitations. The institutions and the participants were all recruited to participate in two RCTs and might not have been representative of nursing home and home-dwelling populations in general. However, the recruitment procedure and inclusion criteria were the same for both patient groups, which made the groups comparable. Furthermore, the participants’ characteristics and level of QoL were in line with findings in other Norwegian studies of persons with dementia [28, 57, 65], indicating that the sample was representative. All home-dwelling PWDs had activities at a day care centre at least once per week, which means that the sample was not representative of the home-dwelling PWD population as a whole. It should be noted that activities at Norwegian day care centres are usually offered to persons who exhibit higher levels or more severe symptoms of dementia, to ease the burden on the family carers.

Validated psychometric questionnaires were used to measure cognitive impairment (CDR) and QoL (QUALID), but proxy assessments always will have less validity than self-assessments. Assessing QoL in PWDs is often done with proxy assessments due to the assumption that the respondents will not able to complete a self-report. However, research has shown that especially those with mild or moderate dementia are capable of completing such reports, and interestingly PWDs in general report that they have a better QoL than their close relatives or care workers do [21, 26]. Therefore, we cannot know whether the scoring in our study truly reflected the participants’ experience, but the QUALID assessment rates concrete and observable behaviour and is a validated tool for this patient group [45]. In our study the patients’ primary nurses scored the psychometric measures, and primary nurses at nursing homes could be expected to have a broad understanding of their patients’ cognitive level, behaviour, and mood. However, the nurses at the day care centre only saw their patients once or twice each week and might not have had the same insights into their patients’ lives. This could have led to bias if the scoring relating to QoL in home-dwelling PWDs was systematically higher than for nursing home PWDs. However, a previous study found no clinically relevant differences in proxy-reported QoL between those in home care and those in institutional long-term care [21].

Actigraphy gives objective data on physical activity. The use of wrist ActiGraphs has been validated for the evaluation of sleep in patients with dementia [66], and the devices have been used to study motor activity patterns in elderly patients with dementia both living in nursing homes and living at home [67, 68]. A specific problem of using actigraphy in this particular patient group could be the removal of the wrist device. We excluded days that had less than 8 h recorded after applying ActiLife’s Wear Time Validation tool (21.75 % of the total number of days); hence, removal of the wrist ActiGraph was not considered a substantial problem. Also, we had to rely on an arbitrary setting of ‘time in bed’, which on the one hand allowed us to obtain a more standardized measure of the amount of sleep during more ‘desirable’ hours of the day or night, but on the other hand did not fully return a measurement of the participants’ actual sleep efficacy. This would have to be taken into account if the findings were to be compared with those in the existing literature.

The model explained 28 % of the variance in QoL, which indicates that other factors not included in the study affected QoL in the patient group. It is reasonable to assume that the PWDs living in nursing homes had poorer health in general, and more comorbid somatic diseases, as might be indicated by the significant differences between the groups in medication and use of walking aids. However, comorbidity and number of diagnoses were not found associated with QoL in another Norwegian study of patients with dementia in nursing homes [31], and earlier research has shown inconsistent associations between medication and QoL in PWDs [21, 31, 40].

Finally, the cross-sectional design of the study restricted the possibility of drawing any conclusions on causality, and the only conclusion that could be derived from the regression model is that living in nursing home is associated with lower QoL. However, the results of the longitudinal analysis conducted on the subgroup population could suggest that residential care contributes in a causal way toward lower QoL.

## Conclusion

The results of our study indicate that living at home as long as possible is not only desirable for economic and/or health political reasons but also is associated with a higher QoL for patients with moderate dementia. More studies are needed to investigate how QoL could be increased for PWDs living in nursing homes.

## Abbreviations

QoL, quality of life; PWDs, persons with dementia; PWD NH, persons with dementia in nursing homes; CDR, The Clinical Dementia Rating scale
